# Uncovering the mechanism of arecoline’s effect on oral submucous fibrosis: integrating transcriptomics and *in vitro* and *in vivo* experiments

**DOI:** 10.3389/fphys.2026.1768602

**Published:** 2026-03-04

**Authors:** Zhenkui Liu, Jian Yi, Fanzuo Zeng, Bowei Chen, Wanling Ning, Jiongwei Tang, Yin OuYang

**Affiliations:** 1 School of Chinese Medicine, Hong Kong Baptist University, Hong Kong SAR, China; 2 The First Hospital of Hunan University of Chinese Medicine, Hunan University of Chinese Medicine, Changsha, Hunan, China; 3 Institute of Traditional Chinese Medicine Resources, Hunan Academy of Traditional Chinese Medicine, Changsha, Hunan, China

**Keywords:** arecoline, experimental verification, hippo signaling pathway, oral submucous fibrosis (OSF), transcriptomics

## Abstract

**Objective:**

This study aimed to elucidate the potential targets and molecular mechanisms underlying arecoline-induced oral submucous fibrosis through integrated transcriptomic profiling and experimental validation.

**Methods:**

Transcriptomic sequencing was first employed to identify key pathways and targets influenced by arecoline in rat oral mucosa and whole blood. Subsequently, *in vitro* experiments using human primary oral mucosal fibroblasts (hOMFs) were conducted to validate the molecular mechanisms.

**Results:**

*In vivo* experiments demonstrated that chronic topical application of arecoline significantly reduced oral opening distance and induced histopathological features of oral submucous fibrosis (OSF), including epithelial atrophy, collagen deposition, and elevated TGF-β expression. Transcriptomic analysis revealed significant enrichment of pathways associated with fibrosis, including PPAR signaling, AMPK signaling, p53 signaling, and Hippo signaling pathways. *In vitro* validation further confirmed that arecoline dose-dependently upregulated α-SMA and Col1a1 expression, enhanced fibroblast proliferation, and activated Hippo pathway effectors (YAP/TAZ).

**Conclusion:**

These findings highlight the Hippo signaling pathway as a critical mediator of arecoline-induced OSF, providing novel insights for therapeutic targeting and mechanistic exploration in OSF management.

## Graphical Abstract

**Figure:**
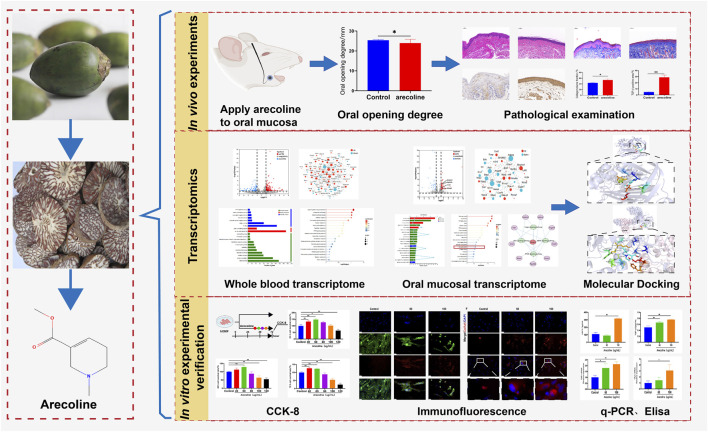

## Highlights

• The initial elucidation of the pathogenic mechanism of oral submucosal fibrosis centered on the Hippo signalling pathway.

• The initial study to explore the mechanism of oral submucosal fibrosis with macro- and micro-level transcriptome analyses of whole blood and mouth mucosal tissue.

## 1 Introduction

Oral submucous fibrosis (OSF) is a chronic, insidious condition marked by submucous fibrosis, resulting in restricted mouth opening and dysphagia, with a considerable risk of progression to malignant oral squamous cell carcinoma (OSCC) (Tilakaratne et al., 2006), hence rendering OSF an important public health concern. Recent epidemiological studies indicate a significant frequency of OSF in regions where betel nut use is prevalent, particularly in South and Southeast Asia, notably in India, Taiwan, and specific provinces of China. The incidence in these regions varies from 0.2% to over 6%, demonstrating a distinct correlation with the intake of betel nut products (Murti et al., 1985; Guo et al., 2021; Warnakulasuriya et al., 2007). In China, betel nut holds a distinctive cultural and historical significance as a material utilised for both medicinal and culinary purposes. It has historically been utilised in traditional Chinese medicine for its properties of alleviating stagnation and eradicating pests (Wang et al., 2024). Despite local acceptance, the international medical community largely contends that betel nut poses detrimental health risks and is classified as a Group 1 carcinogen by the International Agency for Research on Cancer (IARC), leading to numerous countries prohibiting or limiting its use (Li et al., 2023). The disparity between conventional applications and global health issues underscores the pressing necessity for additional investigation into the impact of betel nut on oral health.

Arecoline, the principal bioactive alkaloid in betel nut, has been thoroughly investigated for its involvement in the aetiology of OSF. Arecoline can promote excessive collagen production, impede collagen breakdown, and activate fibroblasts and myofibroblasts, which are essential cellular mediators of fibrosis (Li et al., 2024; Huang et al., 2023). Furthermore, arecoline has demonstrated genotoxic, cytotoxic, and mutagenic effects on oral keratinocytes, undermining epithelial integrity and activating inflammatory and oxidative stress pathways, hence worsening the pathogenesis of OSF (Xu et al., 2020). Despite these advancements, the precise molecular mechanisms and regulatory networks governing OSF remain inadequately comprehended. Current evidence indicates that arecoline influences critical signalling pathways, epigenetic alterations, and extracellular matrix dynamics, necessitating additional investigation (Han et al., 2021; Liu et al., 2024).

Transcriptional analysis, particularly through transcriptomics, offers substantial benefits in the investigation of intricate disorders like OSF. Transcriptomics offers an extensive analysis of gene expression across the entire genome, facilitating the identification of critical molecular pathways, gene networks, and biomarkers linked to illness onset and progression (Zhang et al., 2024). In contrast to conventional approaches, transcriptomics can impartially identify novel genes and regulatory processes potentially unlinked to the disease process. Transcriptomics can elucidate the dynamic molecular characteristics underlying the pathophysiology of OSF by evaluating differential gene expression at distinct disease phases, including modifications in fibrosis-related genes, inflammatory pathways, and cell signalling networks.

This study seeks to investigate the processes via which arecoline induces OSF, emphasizing its impact on the expression of α-sma and Col1a1 in fibroblasts by transcriptomics. This is especially critical due to the escalating burden of OSF in people that consume betel nut and the rising global knowledge of its public health implications.

## 2 Materials and methods

### 2.1 Animals

Eighteen 2-month-old male SPF-grade Sprague-Dawley (SD) rats weighing (200 ± 20) g were purchased from Hunan Slike Jingda Laboratory Animal Co., Ltd. (License No. SCXK [Xiang] 2021–0002; Animal Certification No. 430727241100856613). Animals were housed in the SPF-grade animal facility of the First Affiliated Hospital of Hunan University of Chinese Medicine. Our animal housing conditions included a temperature range of 20 °C–25 °C and relative humidity of 50%–60%. Artificial lighting was alternated between light and dark cycles (12 h light/12 h dark), and animals had free access to food and water. All experiments were approved by the Experimental Animal Ethics Committee of the First Affiliated Hospital of Hunan University of Chinese Medicine (Approval No. ZYFY20230701-001).

### 2.2 Reagents

Arecoline (B74705) was purchased from Yuanye Biotechnology (Shanghai, China). Any of the plant components used in Our study derives from protected or endangered species.The Masson’s Trichrome Staining Kit (G1340) was sourced from Solarbio Science & Technology (Beijing, China). The Phospho-TAZ (Ser66) Antibody (AF4316)and TGF-β antibody (AF1027) was obtained from Affinity Biosciences (Jiangsu, China). The Lamin B1 Rabbit mAb (A11495),YAP1 Rabbit mAb (A19134),phospho-YAP1-S127 Rabbit mAb (AP1398),GAPDH Rabbit mAb (High Dilution) (A19056) and TAZ Rabbit mAb (A23034) was obtained from ABclonal Biotechnology Co., Ltd. (Wuhan, China).The Anti-Fade Mounting Medium with DAPI (G1407) was procured from Servicebio (Wuhan, China). Biotin-conjugated Goat Anti-Rabbit IgG (H + L)(SA00004-2),CoraLite594-conjugated Goat Anti-Rabbit IgG (H + L) (SA00013-4) was acquired from Proteintech (Wuhan, China). alpha-Smooth Muscle Actin (D4K9N) Rabbit Monoclonal Antibody (19245S) and COL1A1 (E8F4L) Rabbit Monoclonal Antibody (72026S) were purchased from Cell Signaling Technology (Boston, MA, USA). NovoStart® SYBR qPCR SuperMix Plus (E096-01A) and NovoScript® Plus All-in-one first Strand cDNA Synthesis SuperMix (gDNA Purge) (E047-01B) were obtained from Novoprotein (Shanghai, China). The Nuclear and Cytoplasmic Protein Extraction Kit (P0027),RNAeasy™ Animal RNA Extraction Kit (Spin Column) (R0026), Actin-Tracker Green-488 (C2201S), immunostaining fixative (P0098), immunostaining permeabilization buffer (P0096), and immunostaining blocking buffer (P0102) were sourced from Beyotime Biotechnology (Shanghai, China). Primers were designed by Wuhan Kingwell Biotechnology Co., Ltd. TGF-β (E-EL-0162) and bFGF (E-EL-H6042) ELISA kits, as well as the Enhanced Cell Counting Kit 8 (WST-8/CCK8) (E-CK-A362), were purchased from Elabscience Biotechnology (Wuhan, China). TRIzol Reagent (15596–026) was obtained from Thermo Fisher Scientific (MA, USA).

### 2.3 *In vivo* experiments

#### 2.3.1 OSF model establishment

The OSF model was established by applying arecoline solution to the left buccal mucosa of rats using cotton swabs for 30 s daily, simulating the oral absorption environment of betel nut chewing, as previously described (Yang et al., 2019). The intervention was maintained for 16 weeks, and model success was confirmed by histopathological examination of oral mucosa. To minimize animal suffering, predefined humane endpoints were established prior to the study. Rats were euthanized if they exhibited any of the following: body weight loss of ≥20% compared with baseline, refusal to eat or drink for 24–48 h, severe reduction in spontaneous activity with inability to access food or water, persistent signs of pain or distress (e.g., hunched posture, vocalization, self-mutilation), marked respiratory distress, or a moribund condition. Animals reaching these humane endpoints were promptly euthanized by overdose of pentobarbital (150 mg/kg) by trained personnel, as approved by the institutional animal care and use committee. Death was confirmed by the absence of spontaneous respiration for at least 5 min, absence of detectable heartbeat on auscultation, lack of corneal reflex, no response to a firm toe pinch, and fixed, dilated pupils. Animals were considered dead only after all these criteria were met.

#### 2.3.2 Experimental grouping and drug administration

Eighteen rats were randomly divided into control and arecoline groups. Based on preliminary studies, the arecoline group received 2 mg/mL arecoline solution applied to the left buccal mucosa, while the control group received an equal volume of distilled water. Applications were performed daily for 30 s over 16 weeks.

#### 2.3.3 Oral opening measurement

Rats were anesthetized with intraperitoneal injection of pentobarbital (50 mg/kg), and a spring dynamometer was used to apply 2 N of opening force to the upper and lower incisors. The passive opening distance between the incisal edges was measured using a vernier caliper. Measurements were repeated three times per animal, and the average value was recorded to the nearest 0.02 mm.

#### 2.3.4 Hematoxylin and eosin (H&E) staining

Left buccal mucosa tissue was collected from three rats, fixed in 4% paraformaldehyde for 24 h, paraffin-embedded, and sectioned coronally for H&E staining. Histopathological damage was observed at ×100 magnification. Images were captured using the Smart Tissue Section Imaging System from PerkinElmer, Inc. in the United States.

#### 2.3.5 Masson’s trichrome staining

Three rats were used in each group to obtain paraffin sections of the buccal mucosa. After dewaxing, the paraffin sections were stained with working staining solution according to the Masson staining kit.Slides were mounted with neutral balsam. Collagen fiber distribution was observed at ×100 magnification, and collagen volume fraction was analyzed using ImageJ software. Images were captured using the Smart Tissue Section Imaging System from PerkinElmer, Inc. in the United States. Before analysis, digital photos were anonymised, tagged, and randomised, with quantitative analysis conducted by researchers blind to group allocations. Three animals were chosen from each group, and non-overlapping visual fields from analogous anatomical locations were collected and analysed; the visual field level data were averaged. Collagen-positive (fibrosis-related) signals were measured as a percentage of the positive region utilising ImageJ/Fiji software. Uniform threshold parameters (Masson: color-based deconvolution segmentation; immunohistochemistry: DAB signal thresholding) were utilised for all photos within the same staining batch, and an identical analytical methodology was employed across all groups.

#### 2.3.6 Immunohistochemical (IHC) staining

Paraffin sections were dewaxed with xylene, ethanol, and hydrated. Antigen retrieval was performed using 1× citrate buffer (pH 6.0), followed by serum blocking at 37 °C for 30 min. Sections were incubated with TGF-β primary antibody (1:200) overnight at 4 °C, washed with PBST, and incubated with secondary antibody at 37 °C for 1 h. DAB chromogenic reaction was performed, followed by hematoxylin counterstaining, dehydration, and mounting. TGF-β expression was observed at ×400 magnification, and ImageJ software was used to select three images of essentially the same location to analyze the positive rate of protein expression. The FIJI version of ImageJ used is a free, open-source Java-based software developed by the National Institutes of Health (NIH). Images were captured using the Smart Tissue Section Imaging System from PerkinElmer, Inc. in the United States.

### 2.4 Transcriptome sequencing and data analysis

Rats were anesthetized with intraperitoneal injection of pentobarbital (50 mg/kg), there underwent a terminal procedure involving the collection of 6–10 mL of blood via the abdominal aorta. Blood samples were collected from the abdominal aorta of control and arecoline-treated rats, mixed with TRIzol reagent, and immediately snap-frozen in liquid nitrogen. The left buccal mucosa was rapidly dissected, snap-frozen in liquid nitrogen, and stored at −80 °C until RNA extraction. Transcriptomic sequencing was performed by Majorbio Bio-Pharm Technology Co., Ltd. (Shanghai, China). Total RNA from buccal mucosa and whole blood was extracted using the 2× CTAB (cetyltrimethylammonium bromide) method. mRNA was isolated from total RNA using Oligo (dT)-conjugated magnetic beads via A-T base pairing with polyA tails. mRNA was fragmented into ∼20 bp segments using fragmentation buffer under optimized conditions. Double-stranded cDNA was synthesized from fragmented mRNA. We employ the “reverse synthesis method” for double-stranded DNA synthesis. The core steps and principles are as follows: RNase H enzyme randomly cleaves the mRNA template in the first-strand product into short fragments. DNA polymerase I is then added. This enzyme uses the RNA fragments from the previous step as primers and synthesizes the second strand using the first-strand cDNA as a template. Simultaneously, its exonuclease activity removes the preceding RNA fragments. Ultimately, DNA fragments synthesized from various starting points are connected into a complete second strand. This forms a blunt-ended double-stranded cDNA with the first strand, ready for subsequent library preparation.

Bioinformatics analysis included the following steps: ① Raw sequence data processing: Quality control of raw reads using FastQC. ② Data filtering: Removal of low-quality reads and adapters using Trimmomatic. ③ Reference genome alignment: Mapping clean reads to the *Rattus norvegicus* reference genome (Rn6) using HISAT2. ④ Expression quantification: Calculation of gene expression levels as read counts using featureCounts. ⑤ Differential expression analysis: Identification of differentially expressed genes (DEGs) with |log2 (fold change)| > 1 and false discovery rate (FDR) < 0.05 using DESeq2.

Functional enrichment analysis was conducted on the Majorbio Cloud Platform (https://cloud.majorbio.com/page/tools.html). Gene Ontology (GO) terms and Kyoto Encyclopedia of Genes and Genomes (KEGG) pathways with FDR <0.05 were considered significantly enriched.

#### 2.4.1 Protein-protein interaction (PPI) network construction

Potential OSF-related targets of arecoline were input into the STRING database (https://string-db.org/) to establish PPI relationships.

#### 2.4.2 Molecular docking

3D structures of target proteins were downloaded from the Protein Data Bank (PDB) and processed in PyMOL 2.5 published and managed by Schrödinger Company to remove water molecules and ligands. 3D structures of compounds were downloaded from PubChem in SDF format, converted to mol2 or PDB format using Open Babel 2.4.1, and docked to target proteins using AutoDock. Docking results were visualized in PyMOL 2.5.

### 2.5 *In vitro* experiments

#### 2.5.1 Cell culture

Primary human oral mucosal fibroblasts (hOMFs) (HUM-iCell-m017) were purchased from Cyagen Biosciences (Shanghai, China). hOMFs were cultured in hOMF complete medium (iCell-m017-002h) at 37 °C in a 5% CO_2_ incubator. All experiments were approved by the Experimental Ethics Committee of the First Affiliated Hospital of Hunan University of Chinese Medicine (Approval No. ZYFY20230701-001).

#### 2.5.2 CCK-8 assay for cell viability

The effects of arecoline on hOMF cell viability were assessed using the CCK-8 assay. Briefly, hOMF cells were seeded in 96-well plates at a density of 1 × 10^5^ cells/mL (100 μL per well). After cell adhesion, the culture medium was replaced with fresh medium containing varying concentrations of arecoline (0, 40, 60, 80, 100, and 120 ug/mL), with six replicate wells per concentration. Following 24 h, 48 h, and 72 h of treatment, 10 μL of CCK-8 reagent was added to each well, and the plates were incubated for 2 h at 37 °C. The optical density (OD) was measured at 450 nm using a microplate reader. Cell viability (%) was calculated as follows: Viability (%) = [(OD treatment −OD blank)/(OD control −OD blank)] × 100%, where *OD blank* represents the background absorbance of wells without cells.

#### 2.5.3 Immunofluorescence staining

Immunofluorescence staining was performed to evaluate the expression of Col1a1 and α-SMA in hOMF cells. Briefly, hOMF cells were seeded in 6-well plates at a density of 3 × 10^5^ cells/mL (1 mL per well). After cell adhesion, the culture medium was replaced with fresh medium containing arecoline at concentrations of 0, 60, or 100 ug/mL. Following 48 h of treatment, cells were fixed with 4% paraformaldehyde, permeabilized with 0.1% Triton X-100, and blocked with 5% bovine serum albumin (BSA). Subsequently, cells were incubated overnight at 4 °C with primary antibodies against Col1a1 (1:200 dilution) and α-SMA (1:400 dilution). After washing, cells were incubated with fluorescent secondary antibodies (1:500 dilution) at 37 °C for 1 h in the dark. Actin cytoskeleton was labeled using Actin-Tracker Green (1:50 dilution) according to the manufacturer’s protocol, followed by nuclear counterstaining with DAPI (5 min at room temperature). Coverslips were mounted onto slides, and fluorescence intensity was quantified using ImageJ software. Images were scanned using a laser scanning confocal microscope from Carl Zeiss in Germany.

#### 2.5.4 ELISA

The levels of TGF-β and bFGF in cell culture supernatants were quantified using a sandwich enzyme-linked immunosorbent assay (ELISA). hOMF cells were seeded in 6-well plates at a density of 1 × 10^6^ cells/mL (1 mL per well). After cell adhesion, the culture medium was replaced with fresh medium containing arecoline at concentrations of 0, 60, or 100 ug/mL. Following 48 h of treatment, supernatants were collected and centrifuged at 1,000 × g for 10 min to remove cellular debris. According to the manufacturer’s instructions, reagents were added to the supernatants, and TGF-β and bFGF levels were measured via a sandwich ELISA. Briefly, standards and samples were added to antibody-precoated wells and incubated at 37 °C for 1 h. After washing, biotinylated detection antibodies were added, followed by horseradish peroxidase (HRP)-conjugated streptavidin. Tetramethylbenzidine (TMB) substrate was used for color development, and the reaction was terminated with stop solution. Optical density (OD) was measured at 450 nm using a microplate reader. The concentrations of TGF-β and bFGF in the samples were calculated based on a standard curve derived from known concentrations of recombinant proteins.

#### 2.5.5 RT-qPCR

hOMFs were seeded in 6-well plates at 1 × 10^6^ cells/mL. After attachment, cells were treated with arecoline (0, 60, or 100 ug/mL) for 48 h. After lysis, washing, and elution of the cell samples, purified RNA samples were obtained. These were then mixed with cDNA Synthesis SuperMix and gDNA Purge, and reverse transcribed into first-strand DNA under the following conditions: 50 °C for 15 min, followed by 85 °C for 5 s. Then analyzed by RT-qPCR for YAP and TAZ mRNA levels. We employed a two-step PCR amplification protocol: 95 °C for 1 min, followed by 40 cycles of 95 °C for 20 s and 60 °C for 1 min. This was followed by a melting curve analysis program: 95 °C for 15 s, 60 °C for 1 min, and 95 °C for 1 s. Our experiments were conducted using a German-made Archimed X4 real-time quantitative PCR instrument. β-actin was used as the internal control, and relative expression was calculated using the 2^−ΔΔCq^ method (Gong et al., 2020). All primers were synthesized by Golden Start Biotechnology Co., Ltd., Wuhan, China. Primer sequences are listed in Table 1.

**TABLE 1:** PCR primer sequences of each gene.

Gene name	Primer sequence (5’∼3′)	Product length
YAP	F:TACTATCAACCAAAGCACC	121
	R:TCCTTCTATGTTCATTCCAT	
TAZ	F:TTCCTAACAGTCCGCCCTAC	131
	R:TTTCCGCATCTCCACAGC	
β-actin	F:TGGACTTCGAGCAAGAGATG	137
	R:GAAGGAAGGCTGGAAGAGTG	

#### 2.5.6 Western bolt

hOMF cells were inoculated in 6-well plates at a concentration of 1 × 10^6^ cells/mL, with 1 mL of cell suspension dispensed into each well. Subsequent to cell attachment, varying doses of arecoline were introduced to the total cell culture media (0 μg/mL, 60 μg/mL, and 100 μg/mL). Following 48 h of intervention, cells were harvested, lysed, and centrifuged to isolate proteins. Nuclear proteins were isolated with the Nuclear and Cytoplasmic Protein Extraction Kit in accordance with the manufacturer’s guidelines. Thereafter, the cells underwent electrophoresis, transfer, blocking, and overnight incubation at 4 °C with primary antibodies YAP (1:20000), *p*-YAP (1:10000), *N*-YAP (1:20000), TAZ (1:1000), *p*-TAZ (1:1000), *N*-TAZ (1:1000), GAPDH (1:20000), and Lamin B1 (1:3000). The following day, a secondary antibody was introduced, and the cells were treated at 37 °C for 1 hour. Protein expression levels of YAP, *p*-YAP, *N*-YAP, TAZ, *p*-TAZ, and *N*-TAZ were assessed utilising ImageJ software. GAPDH served as an internal control for total protein, while Lamin B1 functioned as an internal control for nuclear protein.

### 2.6 Statistical analysis

Statistical analysis was performed using Prism 8.0 software developed by GraphPad Software, Inc. in the United States. All assays were performed in triplicate with three biological replicates and three technical replicates. Normally distributed measurement data are presented as x̄ ± s. One-way analysis of variance (ANOVA) was used to compare means across multiple groups.Data were analyzed using One -way ANOVA followed by Tukey’s multiple comparisons test, with *P* < 0.05 indicating statistical significance.

## 3 Results

### 3.1 OSF induction

**FIGURE 1:**
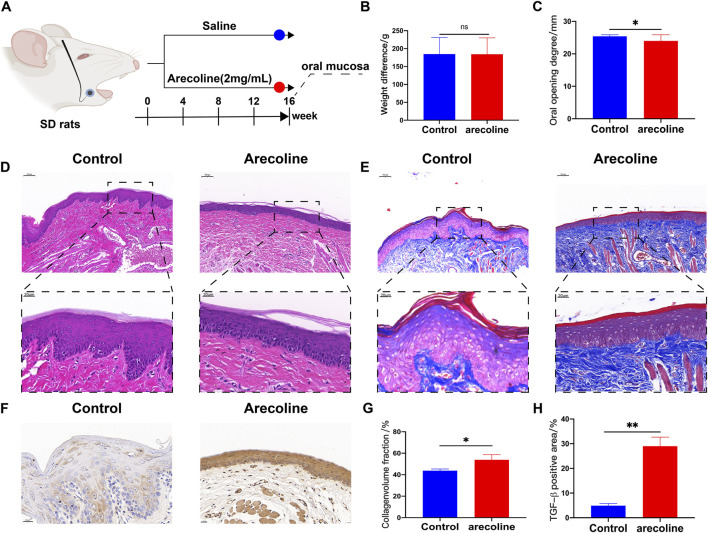
Effects of arecoline on the oral mucosa of SD rats. **(A)** Experimental design flowchart; **(B)** Comparison of body weight changes between the two groups (x̄ ± s, n = 9); **(C)** Comparison of oral opening degrees between the two groups (x̄ ± s, n = 9); **(D)** HE staining of oral buccal mucosa in the two groups, ×100 (n = 3); **(E)** Masson staining of oral buccal mucosa in the two groups, ×100 (n = 3); **(F)** TGF-β immunohistochemistry of oral buccal mucosa in the two groups, ×400 (n = 3); **(G)** Analysis of collagen volume fraction in oral buccal mucosa of the two groups (x̄ ± s, n = 3); **(H)** Analysis of the positive rate of TGF-β expression in oral buccal mucosa of the two groups (x̄ ± s, n = 3). Compared with the Control group, *P < 0.05, **P < 0.01.

In this study, we induced the OSF model by applying arecoline to the oral buccal mucosa. The establishment of the OSF model was evaluated by measuring the oral opening degree, pathological examination, and immunohistochemistry in rats (Figure 1A). After 16 weeks of intervention, there was no significant difference in the average body weight gain between the arecoline group and the control group (*P* > 0.05) (Figure 1B). Compared with the control group, the oral opening degree of rats in the arecoline group was reduced, and immunohistochemistry showed a significant increase in the expression of TGF-β in the buccal mucosa (*P* < 0.05 or *P* < 0.01) (Figures 1C,F,H).

HE and Masson staining results showed that the buccal mucosa tissue in the control group was arranged neatly with a normal structure. In contrast, the buccal mucosa epithelium of rats in the arecoline group was atrophied and thinned, the lamina propria was significantly thickened, collagen fibers were arranged in a disordered and wavy shape, the number of blood vessels was reduced, and hyaline-like degeneration and cellular vacuolation were observed. Additionally, there was an accumulation of collagen fibers and an increase in the collagen volume fraction in the buccal mucosa (*P* < 0.05) (Figures 1D,E,G).

### 3.2 RNA-seq analysis of arecoline-induced OSF based on whole blood

**FIGURE 2:**
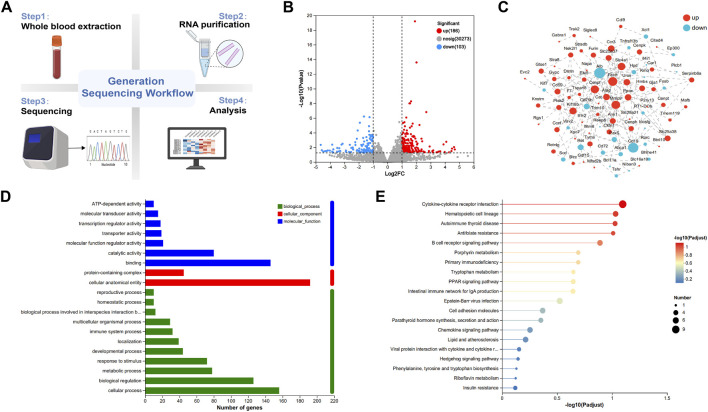
RNA-seq analysis of arecoline-induced OSF based on whole blood. **(A)** Experimental design flowchart (n = 3); **(B)** Volcano plot of DEGs; **(C)** PPI network of DEGs; **(D)** GO functional analysis chart; **(E)** KEGG enrichment analysis chart.

Whole blood was collected from rats for transcriptomics sequencing (Figure 2A). Using whole blood transcriptomics, 30,562 potential targets were identified. By applying the criteria of |log2(FC)| > 1 and FDR p.adj <0.05 for screening differentially expressed genes (DEGs), a total of 289 DEGs were identified, including 186 downregulated DEGs and 106 upregulated DEGs (Figure 2B). These 289 DEGs were imported into the STRING database (https://string-db.org/) to construct a protein-protein interaction (PPI) network. The obtained data were downloaded in TSV format and imported into Cytoscape 3.9.2 software for visualization and analysis (Figure 2C). The node area represents the degree value of the node. The larger the node area, the greater the corresponding degree value, indicating a more important node, such as Alb, Fech, Cenpf, Mmp9, Slc4a1, etc.

The DEGs were entered into the Majorbio Cloud Platform for GO functional enrichment and KEGG pathway enrichment analysis. The top 20 GO functional enrichment bubble charts, including cellular component, molecular function, and biological process, and KEGG pathway enrichment bubble charts were plotted in ascending order of FDR. The GO analysis results showed that biological process (BP) related entries mainly involved cellular processes, biological regulation, metabolic processes, etc.,; cellular component (CC) related entries mainly involved protein-containing complexes, cellular anatomical entities, etc.,; molecular function (MF) related entries mainly involved binding, catalytic activity, molecular function regulator activity, etc. (Figure 2D). Furthermore, KEGG pathway enrichment analysis of core genes indicated that the pathogenesis of arecoline-induced OSF mainly involved cytokine-cytokine receptor interaction, PPAR signaling pathway, hedgehog signaling pathway, etc. (Figure 2E).

### 3.3 RNA-seq analysis of arecoline-induced OSF based on oral mucosa

**FIGURE 3:**
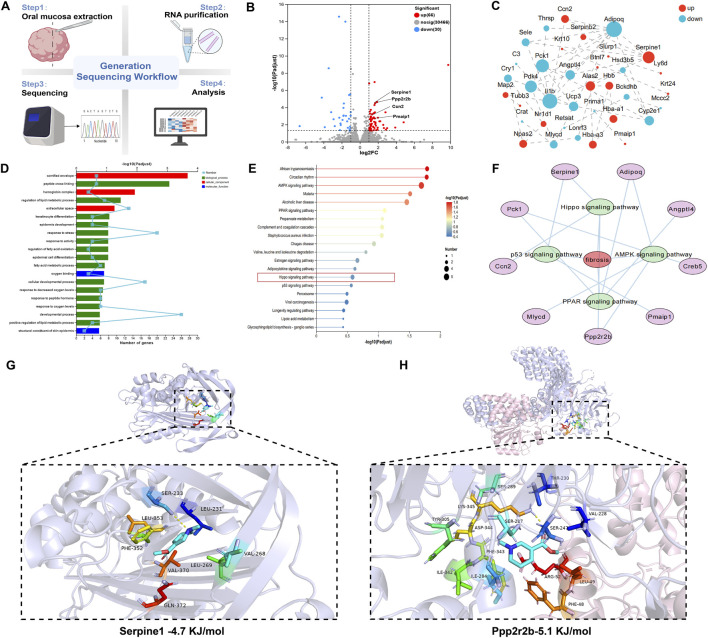
RNA-seq analysis of arecoline-induced OSF based on oral mucosa. **(A)** Experimental design flowchart (n = 3); **(B)** Volcano plot of DEGs; **(C)** PPI network of DEGs; **(D)** GO functional analysis chart; **(E)** KEGG enrichment analysis chart; **(F)** Disease-target-pathway network diagram; **(G)** Molecular docking diagram of arecoline and Serpine1,The core hydrogen bond has SER-233,LEU-231; **(H)** Molecular docking diagram of arecoline and Ppp2r2b,The core hydrogen bond has LYS-345,SER-242.

Oral mucosa was collected from rats for transcriptomics sequencing (Figure 3A). Using transcriptomics, 30,562 potential targets were identified. By applying the criteria of |log2(FC)| > 1 and FDR p.adj <0.05 for screening DEGs, a total of 96 DEGs were identified, including 30 downregulated DEGs and 66 upregulated DEGs (Figure 3B). These 96 DEGs were imported into the STRING database (https://string-db.org/) to construct a PPI network. The obtained data were downloaded in TSV format and imported into Cytoscape 3.9.2 software for visualization and analysis (Figure 3C). The node area represents the degree value of the node. The larger the node area, the greater the corresponding degree value, indicating a more important node, such as Adipoq, Serpine1, ll1b, Pck1, Pdk4, etc.

The DEGs were entered into the Majorbio Cloud Platform for GO functional enrichment and KEGG pathway enrichment analysis. The top 20 GO functional enrichment bubble charts, including cellular component, molecular function, and biological process, and KEGG pathway enrichment bubble charts were plotted in ascending order of FDR. The GO analysis results showed that biological process (BP) related entries mainly involved peptide cross-linking, regulation of lipid metabolic process, keratinocyte differentiation, epidermis development, etc.,; cellular component (CC) related entries mainly involved oxygen binding, structural constituent of skin epidermis, etc.,; molecular function (MF) related entries mainly involved cornified envelope, hemoglobin complex, etc. (Figure 3D). Furthermore, KEGG pathway enrichment analysis of core genes indicated that the pathogenesis of arecoline-induced OSF mainly involved AMPK signaling pathway, PPAR signaling pathway, p53 signaling pathway, Hippo signaling pathway, etc. (Figure 3E).

Based on the results of KEGG pathway enrichment analysis, a disease-target-pathway network diagram was constructed (Figure 3F). The pathways related to arecoline-induced OSF included AMPK signaling pathway, PPAR signaling pathway, p53 signaling pathway, and Hippo signaling pathway. The targets involved in these pathways were Adipoq, Serpine1, Ccn2, Pck1, Ppp2r2b, etc.

According to relevant literature reports, the pathogenic mechanism of OSF may be related to the Hippo pathway. Molecular docking was performed between arecoline and two targets (Serpine1, Ppp2r2b) related to the Hippo pathway (Figures 3G,H). A binding energy <−5.0 kJ/mol indicates strong affinity activity. Our results showed that the binding energy between arecoline and Ppp2r2b was less than −5.0 kJ/mol, indicating that arecoline has a good affinity with targets related to the Hippo pathway, suggesting that arecoline may induce OSF through the Hippo pathway.

### 3.4 *In vitro* experimental verification

**FIGURE 4:**
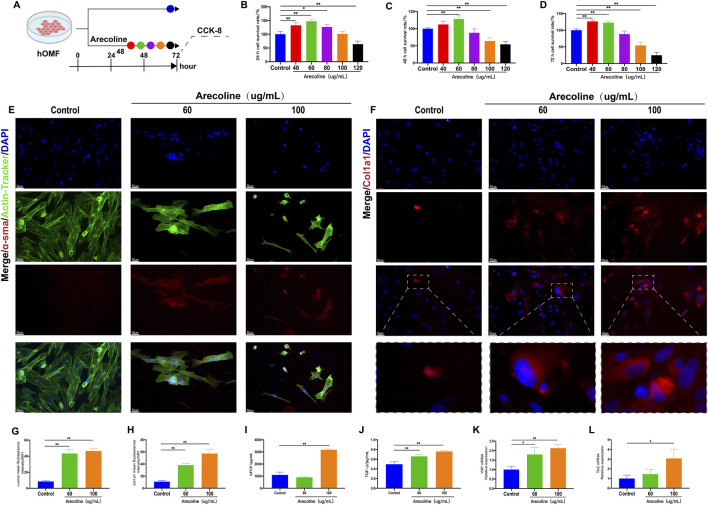
Arecoline-induced fibrosis in hOMF cells. **(A)** Experimental design flowchart; **(B)** Effects of different doses of arecoline on hOMF cell viability at 24h ( $\bar{x}$ ±s, n = 3); **(C)** Effects of different doses of arecoline on hOMF cell viability at 48h ( $\bar{x}$ ±s, n = 3); **(D)** Effects of different doses of arecoline on hOMF cell viability at 72h ( $\bar{x}$ ±s, n = 3); **(E)** Immunofluorescence staining of α-SMA expression in hOMF cells treated with arecoline (n = 3); (200x); **(F)** Immunofluorescence staining of Col1a1 expression in hOMF cells treated with arecoline (n = 3); (200x); **(G)** Quantitative analysis of α-SMA fluorescence intensity ( $\bar{x}$ ±*s*, n = 3); **(H)** Quantitative analysis of Col1a1 fluorescence intensity ( $\bar{x}$ ±s, n = 3). **(I)** Protein expression levels of bFGF ( $\bar{x}$ ±s, n = 3); **(J)** Protein expression levels of TGF-β1 ( $\bar{x}$ ±s, n = 3); **(K)** Relative mRNA expression levels of YAP ( $\bar{x}$ ±s, n = 3); **(L)** Relative mRNA expression levels of TAZ ( $\bar{x}$ ±s, n = 3). Compared with the Control group, *P < 0.05, **P < 0.01.

The biological activity of arecoline on hOMF cells was studied using the CCK-8 method (Figure 4A). The results showed that compared with the control group, low-dose arecoline promoted the proliferation of hOMF cells. As the concentration of arecoline increased, it caused varying degrees of damage to hOMF cells (*P* < 0.05 or *P* < 0.01) (Figures 4B–D). Human investigations indicated peak arecoline concentrations in saliva during mastication, ranging from 5.66 to 97.39 μg/mL, which coincides with our dosage range (Cox et al., 2010).Therefore, subsequent experiments used 60 ug/mL and 100 ug/mL arecoline.

α-SMA and Col1a1 are two key markers and effector molecules in the fibrosis process. To evaluate the degree of fibrosis induced by arecoline-activated hOMF cells, the expression of α-SMA and Col1a1 in hOMF cells after arecoline administration was detected using immunofluorescence. The results showed that compared with the control group, the expression of α-SMA and Col1a1 was significantly increased with the increase of arecoline concentration (*P* < 0.01) (Figures 4E–H).

bFGF (basic fibroblast growth factor) and TGF-β1 (transforming growth factor β1) play important roles in the fibrosis process, mainly by regulating cell proliferation, differentiation, and the synthesis and degradation of extracellular matrix (ECM) to promote the occurrence and development of fibrosis. To further evaluate the expression of related growth factors during arecoline-activated hOMF cell-induced fibrosis, the expression of bFGF and TGF-β1 in hOMF cells after arecoline administration was detected using ELISA kits. The results showed that compared with the control group, the expression of TGF-β1 was significantly increased with the increase of arecoline concentration (*P* < 0.01). After administration of 100 ug/mL arecoline, the expression of bFGF was significantly increased (*P* < 0.01) (Figures 4I,J).

**FIGURE 5:**
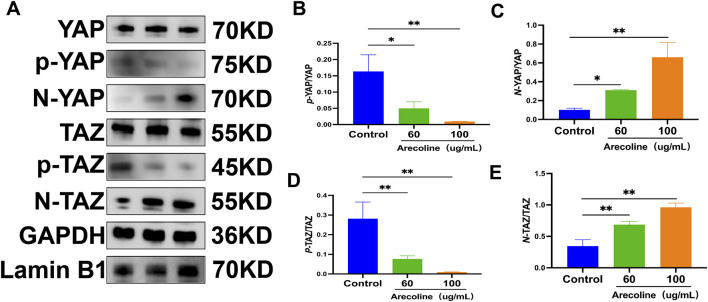
Impact of arecoline on YAP/TAZ protein expression in hOMF cells. **(A)** Representative Western blot bands; **(B)** Relative quantification of *p*-YAP protein in each group ( $\bar{x}$ ±s, n = 3); **(C)** Relative quantification of N-YAP protein in each group ( $\bar{x}$ ±s, n = 3); **(D)** Relative quantification of p-TAZ protein in each group ( $\bar{x}$ ±s, n = 3); **(E)** Relative quantification of N -TAZ protein in each group ( $\bar{x}$ ±s, n = 3); Compared to the Control group, *P < 0.05, **P < 0.01.

To further explore whether arecoline-induced OSF is related to the activation of the Hippo pathway, this study examined the mRNA expression levels of important effector factors and downstream YAP and TAZ in this pathway. Compared with the control group, the mRNA levels of *YAP* and *TAZ* were significantly increased with the increase of arecoline concentration (*P* < 0.05 or *P* < 0.01) (Figures 4K,L). Moreover, Western blot analysis indicated that, relative to the control group, a rise in arecoline concentration resulted in decreased expression levels of *p*-YAP and *p*-YAP proteins, while the nuclear translocation levels of YAP(*N*-YAP) and TAZ (*N*-TAZ) were considerably elevated (*P* < 0.05 or *P* < 0.01) (Figure 5).

## 4 Discussion

In this study, we first investigated the effects of arecoline on the oral mucosa of rats. The results showed that arecoline could reduce the mouth opening of rats, damage the oral mucosa of rats, and cause oral submucosal fibrosis. In addition, we explored the potential molecular mechanism of arecoline-induced OSF by integrated transcriptomics and *in vitro* experiments and focused on its possible regulation of fibroblast function through the Hippo signaling pathway, ultimately promoting the occurrence and development of fibrosis. Our results showed that arecoline is an important compound that induces OSF, and the Hippo signaling pathway is a key pathway for arecoline to cause OSF, and Serpine1 and *Ppp2r2b* may be key molecules. PPI network analysis showed that genes such as *Alb*, *Fech*, *Cenpf*, *Mmp9*, and *Slc4a1* had high centrality in whole blood samples, while genes such as *Adipoq*, *Serpine1*, *ll1b*, *Pck1*, and *Pdk4* occupied an important position in oral mucosal samples.

OSF is largely defined by localised oral fibrosis; nevertheless, exposure to arecoline and the fibrotic process frequently coincide with changes in systemic metabolic, inflammatory, and fibrinolytic systems. Peripheral blood can indicate the body’s systemic responses regarding stress, immunity, and metabolism, hence offering insights into underlying factors related to local lesions (Zhao et al., 2024; Tang et al., 2025). Moreover, the oral mucosa serves as a primary target tissue of OSF, with its transcriptional alterations more accurately reflecting the milieu and intercellular interactions associated with fibrosis. The interplay of these two elements facilitates the formation of a more comprehensive pathogenic chain at both the systemic and local microenvironment levels. Based on this, this study also included transcriptomic analysis of whole blood and oral mucosal tissues. We identified 289 and 96 DEGs from transcriptome data of rat whole blood and oral mucosal tissues, respectively. Numerous genes, including *Adipoq*, *Serpine1*, *Pck1*, and *Pdk4*, exhibited substantial alterations in both tissues.Studies have shown that *Adipoq* plays an important role in metabolic regulation, anti-inflammatory and anti-fibrosis processes. Its downregulation may lead to enhanced inflammatory response, thereby promoting tissue fibrosis (Gong et al., 2020; Sakane et al., 2021). In addition, there are studies that have shown that *Serpine1* can regulate the process of fibrosis, and its upregulation is positively correlated with the degree of tissue fibrosis (Quan et al., 2024). Our study also confirmed that arecoline can cause *Adipoq* downregulation, and *Serpine1* was highly enriched in this study, and molecular docking results showed that arecoline has a strong binding affinity with it, which is consistent with previous transcriptional studies. Therefore, it can be further explained that arecoline is an important inducing factor of OSF, and *Serpine1*may be one of the key molecules for arecoline-induced OSF.

Further KEGG pathway enrichment analysis showed that arecoline may mediate the occurrence and development of OSF through multiple signaling pathways, including cytokine-cytokine receptor interaction, PPAR signaling pathway, AMPK signaling pathway, p53 signaling pathway, Hippo signaling pathway, etc. In the pathogenesis of OSF, PPAR, AMPK and p53 signaling pathways may play an important role. The PPAR signaling pathway is mainly involved in the regulation of lipid metabolism, and the occurrence of OSF is usually accompanied by metabolic abnormalities, suggesting that this pathway may play a certain role in the pathological process of OSF induced by arecoline (Christofides et al., 2021). The AMPK signaling pathway plays a key regulatory role in energy metabolism and cell stress response. Studies have shown that AMPK plays an important role in a variety of tissue fibrosis diseases, and its activation may help slow down the progression of fibrosis,and Studies indicates that mesenchymal stem cell exosomes can target the AMPK system and impede OSF progression. (Palomer et al., 2025; Liu et al., 2021). The p53 signaling pathway plays a core role in cell cycle regulation and apoptosis. Its abnormal activation may lead to a decrease in cell apoptosis rate, thereby promoting abnormal proliferation of fibroblasts and continuous deposition of ECM (Liu et al., 2023). This study found that these signaling pathways were significantly affected by arecoline, further supporting the mechanism of action of arecoline as an OSF-inducing factor.

Among the numerous enriched pathways, we ultimately concentrated our research on the Hippo signalling pathway. The Hippo pathway plays a key role in cell proliferation, differentiation, and apoptosis, and is closely related to tumorigenesis and tissue fibrosis. Related studies have shown that the Hippo signaling pathway plays an important role in various fibrotic diseases such as liver fibrosis, pulmonary fibrosis, and myocardial fibrosis (Shen et al., 2022). Its crucial function in the fundamental axis of fibrosis”cellular mechanosensory perception—cell fate determination—matrix deposition” is well aligned with the pathogenic mechanism of OSF. Moreover, systematic evidence about the Hippo route in OSF is somewhat inadequate, and investigations into the “arecoline-Hippo-fibroblast activation” pathway are still scarce. This study aims to clarify the Hippo pathway as a significant mechanism for arecoline-induced OSF, based on transcriptome enrichment results and *in vitro* experimental confirmation, offering innovation and additional value.In order to further verify the effect of arecoline on the Hippo pathway, we performed molecular docking analysis on *Serpine1* and *Ppp2r2b*. The molecular docking results showed that the binding energy between arecoline and *Ppp2r2b* was less than −5.0 kJ/mol, indicating that there was a strong affinity between the two. Molecular docking data indicate that arecoline may interact with Serpine1 and *Ppp2r2b*, offering structural insights supporting our hypothesis that “arecoline may influence critical nodes in the Hippo pathway”. In addition, cell experiments further confirmed that arecoline can significantly induce changes in the expression of Hippo pathway-related molecules, enhance fibroblast activity, and accelerate the process of fibrosis. In our *in vitro* tests, we established the concentration of arecoline to be 60–100 μg/mL via a CCK-8 assay, which corresponds to roughly 0.39–0.64 mM based on the molecular weight of arecoline. Human studies indicate that arecoline content in saliva can attain levels of up to 140 μg/mL (about 0.9 mM) after areca nut mastication (Chang et al., 1999). Prior research has demonstrated that arecoline at elevated millimolar concentrations (>0.8 mM) exhibits considerable cytotoxicity towards oral/buccal fibroblasts (Chang et al., 2000), and the maximum dosage we selected remains within the established upper limit of saliva exposure. Given that *in vitro* exposure is typically continuous, whereas human exposure when chewing is intermittent (“pulsed”), subsequent research will implement a pulsed exposure methodology and directly quantify arecoline levels in saliva, plasma, and tissue to refine *in vitro* experimental parameters.

This further suggests that it may play a key role in the occurrence and development of OSF. It is important to recognise that OSF is not a disease process driven by a single cell type; its progression entails the synergistic influence of various cells and components of the microenvironment, including epithelial cells, endothelial cells, and fibroblasts. This study selected oral mucosal fibroblasts as the primary *in vitro* model due to their pivotal role as effector cells in extracellular matrix deposition and fibrotic phenotype development, as well as their suitability for directly evaluating the effects of arecoline on cell viability, fibrosis-related gene expression, and matrix remodelling. Future research should incorporate multi-cell co-culture systems, organoid models, or somatic cell lineage tracing/single-cell omics to more accurately reconstruct the cell interaction network within the OSF microenvironment and elucidate the respective contributions of various cell populations to arecoline-induced fibrosis.

This research possesses multiple limitations. Initially, while we noted that arecoline can alter the expression of molecules associated with the Hippo system, resulting in an exacerbated fibrotic phenotype, rescue investigations employing specific inhibitors or agonists of the Hippo pathway have not been performed to yet. Consequently, causal evidence about whether alterations in the Hippo pathway are a requisite condition for arecoline-induced fibrosis is still insufficient. Future research will focus on establishing more robust causal chain validation using pharmacological interventions and genetic techniques, while further clarifying the regulatory interactions of upstream mechanotransduction, inflammatory variables, and metabolic signals on the Hippo axis. Secondly, molecular docking is a hypothetical assessment and cannot currently supplant direct binding and functional confirmation. Future study will necessitate a multi-tiered experimental methodology (binding assays, interaction validation, and *in vitro*/*in vivo* functional interventions) to systematically validate potential nodes such as *Serpine1* and *Ppp2r2b*. Moreover, the limited quantity of replicate samples in the omics data of this study may diminish the statistical power of differential expression analysis and heighten the likelihood of false positives. However, we validated the model’s reliability through pathological testing before transcriptome analysis to maximize the robustness and reproducibility of the results. However, additional validation in separate cohorts with larger sample numbers and increased biological replicates is necessary to enhance the reliability of our conclusions.

In summary, this study systematically revealed the potential molecular mechanism of arecoline-induced OSF through transcriptomics and *in vivo* and *in vitro* experiments. The study found that arecoline can affect fibroblast activation and ECM deposition by regulating Hippo signaling pathway-related genes *Serpine1* and *Ppp2r2b*, and promote the occurrence of oral mucosal fibrosis. In addition, the participation of signaling pathways such as PPAR, AMPK, and p53 also provides a new research direction for the pathogenic mechanism of OSF. Future studies can further explore the specific mechanism of action of the Hippo signaling pathway in OSF and try to develop potential therapeutic strategies for this pathway, in order to provide new theoretical basis and targeted intervention methods for the prevention and treatment of OSF.

## Data Availability

The datasets presented in this study can be found in online repositories. The names of the repository/repositories and accession number(s) can be found below: https://www.ncbi.nlm.nih.gov/.
